# Electrothermally-Actuated Micromirrors with Bimorph Actuators—Bending-Type and Torsion-Type

**DOI:** 10.3390/s150614745

**Published:** 2015-06-22

**Authors:** Cheng-Hua Tsai, Chun-Wei Tsai, Hsu-Tang Chang, Shih-Hsiang Liu, Jui-Che Tsai

**Affiliations:** Graduate Institute of Photonics and Optoelectronics and the Department of Electrical Engineering, National Taiwan University, Taipei 10617, Taiwan; E-Mails: chenghua.tsai@gmail.com (C.-H.T.); d98941019@ntu.edu.tw (C.-W.T.); ckjamesbond@gmail.com (H.-T.C.); r01941068@ntu.edu.tw (S.-H.L.)

**Keywords:** microelectromechanical devices, electrothermal effects, silicon on insulator

## Abstract

Three different electrothermally-actuated MEMS micromirrors with Cr/Au-Si bimorph actuators are proposed. The devices are fabricated with the SOIMUMPs process developed by MEMSCAP, Inc. (Durham, NC, USA). A silicon-on-insulator MEMS process has been employed for the fabrication of these micromirrors. Electrothermal actuation has achieved a large angular movement in the micromirrors. Application of an external electric current 0.04 A to the bending-type, restricted-torsion-type, and free-torsion-type mirrors achieved rotation angles of 1.69°, 3.28°, and 3.64°, respectively.

## 1. Introduction

Micro-electro-mechanical systems (MEMS)-based micromirrors are widely used for various applications, including optical communication [1], optical switches [2], optical displays [3], biomedical imaging [4,5], and optical interconnects [6]. Proposed driving mechanisms for MEMS mirrors include electrostatic, magnetic, piezoelectric, and thermal actuation. The major advantage of electrostatic actuation is the simple capacitor-like structures [7,8,9]. However, a high driving voltage is generally required. Magnetic actuation usually causes a large linear or angular displacement. There are two common approaches to implement magnetic actuation. One is to utilize the Lorentz force [10]; this requires feeding an electric current into the device, resulting in constant power dissipation due to ohmic heating. The other is to make the entire or part of the device out of a magnetic material, and use an external magnet to actuate the device [11,12]. Regarding piezoelectric actuation [13], piezoelectric materials are relatively less common in semiconductor processes. Thermal actuation can generally achieve relatively large displacement and rotation. The structures can be heated by applying a current. Although thermal actuation normally requires constant power dissipation and may be unable to operate at very high frequencies, its large rotation angle with low driving voltage make it ideal for other applications [14,15,16,17,18]. This study will compare mirrors with different thermal bimorph actuators. A bimorph actuator deforms when being heated due to the mismatch in the coefficients of thermal expansion (CTE) [19,20,21,22].

Thanks to its process maturity, SOI processes are commonly used to fabricate electrothermal actuators [23]. Particularly, several electrothermal actuators manufactured with standard foundry SOI processes such as the SOIMUMPs process (MEMSCAP Inc., Durham, NC, USA) were reported [24,25,26].

Generally, in an electrothermally actuated tilting mirror, the bimorph structures serve as the bending flexures [27]. However, it is more straightforward to accomplish mirror tilt using torsion motion because the rotation axis can be easily defined. Therefore, in this paper, we present three different MEMS mirrors that can be electrothermally-actuated; in addition to a bending-type mirror, we also demonstrate mirrors in which the bimorph structures serve as the torsion beams. All the devices are fabricated with the SOIMUMPs process. There is a great potential for mass production and monolithic integration with other MEMS components.

## 2. Design and Fabrication

**Figure 1:**
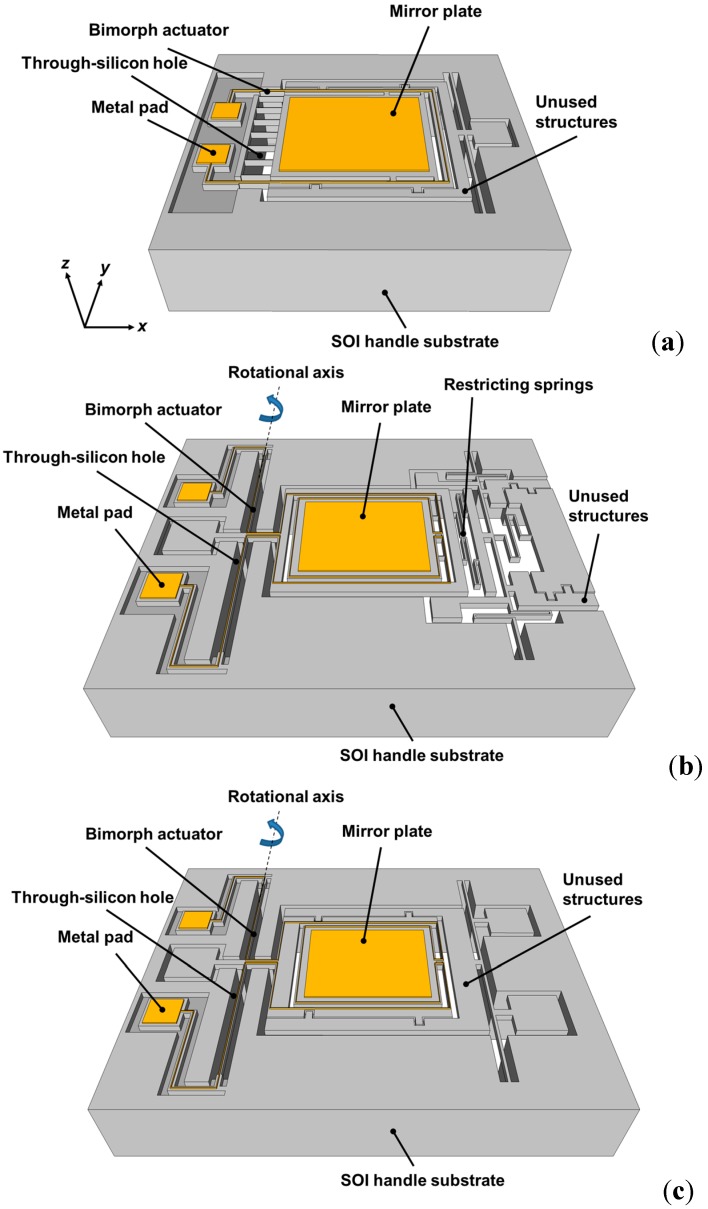
The 3-D schematic drawings of the (**a**) bending-type, (**b**) restricted-torsion-type and (**c**) free-torsion-type electrothermally-actuated micro-electro-mechanical systems (MEMS) mirrors. For simplicity, buried oxide is not shown.

Figure 1 shows the 3-D schematic drawings of the electrothermally-actuated MEMS mirrors. Three different devices were designed: ‘bending-type mirror’ (Figure 1a), ‘restricted-torsion-type mirror’ (Figure 1b), and ‘free-torsion-type mirror’ (Figure 1c). The ‘bending-type’ mirror has two bimorph actuators on the upper left and lower left; the left side of the mirror connects through seven bending beams with the Si substrate. The widths of the bimorph actuator and the bending beam are 10 and 4 μm, respectively. The bimorph actuator and bending beam are both 150 μm long. The resonance frequency of the ‘bending-type’ mirror is estimated as 28,597 Hz. As for the ‘restricted-torsion-type’ mirror, its one end is connected to a torsion spring, and the other end is restricted by some springs. The length and width of the torsion spring are 925 and 16 μm, respectively. The resonance frequency of each type of mirror is calculated. It can be observed that in addition to the two bimorph actuators, the bending-type mirror has seven additional bending beams connecting to the anchor, so it is expected to have higher resonance frequency than the others. Ignoring the restricting springs, the resonance frequency of the ‘restricted-torsion-type’ mirror is estimated as 835.6 Hz. In a ‘free-torsion-type’ mirror, only one end of mirror is connected to a torsion spring. The length and width of the torsion spring are 925 and 16 μm, respectively. The resonance frequency of the ‘free-torsion-type’ mirror is estimated as 848.7 Hz. The overhead views of the three bimorph actuator designs show that the Cr/Au-Si bimorph structures overlap with a through-silicon hole below them. Table 1 shows the dimensions and parameters of the three proposed mirror designs. The mass of the three mirrors, including frame and the metal coating, is 2.37 × 10^−5^, 3.31 × 10^−5^, and 3.24 × 10^−5 g, respectively^. The resonance frequencies are estimated values for devices without metal. Besides, the radius of curvature (R) of the fabricated mirror with metal coating is measured to be ~20 mm.

**Table 1:** Dimensions/parameters for three micromirror designs.

Material	Bending-Type	Restricted-Torsion-Type	Free-Torsion-Type	Unit
Overall footprint	1.85 × 1.73	2.68 × 2.36	2.81 × 2.36	mm^2^
Mirror plate	0.91 × 0.91	1.13 × 1.02	1.10 × 1.02	mm^2^
Gold-coated area	760 × 670	780 × 680	780 × 680	μm^2^
Thicknesses of the silicon device layer and the handle substrate	10, 400	10, 400	10, 400	μm
Bimorph actuator length and width	150, 10	925, 16	925, 16	μm
Mass of the mirror including frame and the metal coating	2.37 × 10^−5^	3.31 × 10^−5^	3.24 × 10^−5^	g
Resonance frequency	28597	835.6	848.7	Hz

Each device consists of a gold-coated mirror area, metal pads for landing the probes, and bimorph actuators. The bimorph actuator consists of a two-layer structure in which each layer has different thermal expansion coefficients; A Cr/Au electrically conducting layer is on top of the Si beam suspended over a through-silicon hole. In the torsion-type mirrors, the bimorph beam is also the torsion spring of the mirror plate. Thermal stress causes the torsion spring to deform. Table 2 shows the material properties of the bimorph actuators.

**Table 2:** Material properties of the bimorph actuator.

Material	Silicon [28,29]	Cr [29]	Au [29]	Unit
Coefficient of thermal expansion (CTE) α	2.6	4.9	14.2	10^−6^/°C
Young’s modulus *E*	160	279	79	GPa
Thermal conductivity *K*	149	93.9	318	W/(m∙°C)
Mass Density ρ	2.33	7.14	19.3	g/cm^3^
Poisson’s ratio ν	0.28	0.21	0.44	

The bimorph actuator is composed of a Cr/Au top layer and Si bottom layer. Metal materials such as Cr and Au have a higher CTE compared to silicon. The deformation of the bimorph actuator during heating results from the CTE mismatch between layers. The devices are fabricated with the SOIMUMPs process developed by MEMSCAP, Inc. (Durham, NC, USA) [30], which uses silicon-on-insulator (SOI) wafers with a 10-μm device layer, 1-μm buried oxide, and 400-μm handle substrate. In the region of the mirror plate, a metal layer of 50-nm Cr and 600-nm Au is coated on the top of the device layer to increase the optical reflectivity. At the end of the fabrication process, the mirror plate and bimorph actuators are released.

**Figure 2:**
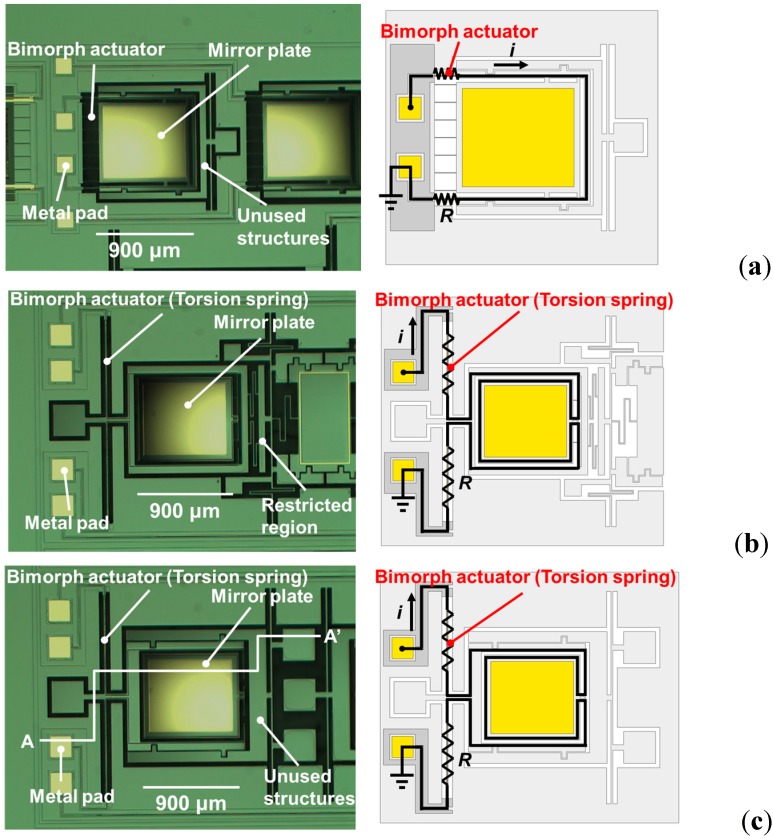
Photos of the three different electrothermally-actuated MEMS mirrors: (**a**) bending-type, (**b**) restricted-torsion-type, and (**c**) free-torsion-type.

**Figure 3:**
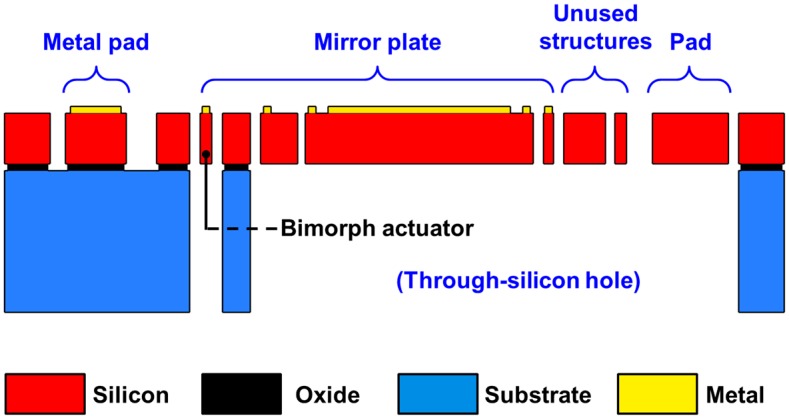
Schematic cross-section view along A–A’ of Figure 2c. The structure details are depicted and the materials used are shown.

Figures 2a–c are optical microscope images of the bending-type mirror, restricted-torsion-type mirror, and free-torsion-type mirror, respectively. Figure 3 is a schematic drawing of the device from a cross-sectional view along the A–A’ shown in Figure 2c. The structure details are depicted and the materials used are shown. These mirrors are actuated by sending a current mainly through the Cr/Au top layer. The temperature of the bimorph actuator is then substantially increased by Joule heating. The Cr/Au top layer has a higher effective CTE compared to Si bottom layer, causing the top layer expands differently from the bottom layer. This further causes the mirror to tilt away from its initial posture.

## 3. Experimental Section

**Figure 4:**
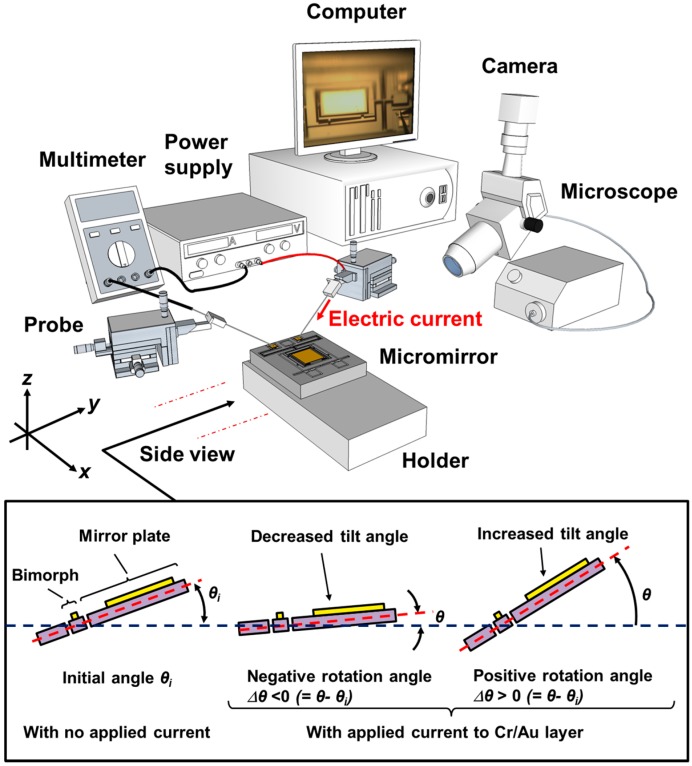
Experimental setup for measuring the rotation angle, corresponding voltage and electrical resistance of the MEMS mirror when an external electric current is applied.

Figure 4 shows the experimental setup used to determine how the applied electric current affects the rotation angle, where the microscope helps with observing the mirror motion. The power supply, current meter, probe and the device are connected in series. The initial pre-tilt angle relative to the substrate surface, if any, is defined as θ_i_. The resolution is better than 0.02°. When applying the current, in the case where the mirror tilt angle relative to the substrate surface, θ, is greater than the pre-tilt angle θ_i_, the rotation angle Δθ (= θ−θ_i_) is positive. In contrast, if the mirror tilt angle relative to the substrate surface, θ, is less than the initial pre-tilt angle, the rotation angle Δθ (= θ−θ_i_) is negative. For each design, we test two identical devices, but with different total electrical resistances due to their difference in total wire length; the theoretical resistance values are calculated to be approximately 65 and 150 Ω, respectively, ignoring the underlying silicon. Later in the paper, we will use ‘65 Ω’ and ‘150 Ω’ to denote the low-resistance and high-resistance devices in each design, respectively. During testing, the chip is mounted on a fixed holder. After applying an electric current to the device through the probes, the rotation angle, corresponding voltage and electrical resistance are measured. The bending-type, restricted-torsion-type, and free-torsion-type mirrors are then compared.

**Figure 5:**
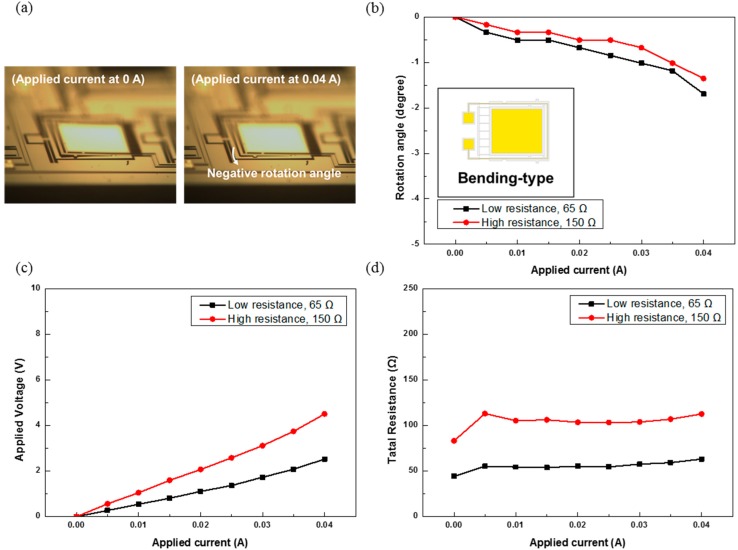
Experimental results for the bending-type MEMS mirrors of different wire resistances: (**a**) optical microscope images; (**b**) rotation angles; (**c**) applied voltages; and (**d**) total resistances.

Figure 5 shows the experimental results for the bending-type MEMS mirrors of different wire resistances. The total resistance including that between the current input and output pads and that of external wires and components is also measured. When the applied current increases, the resistance is substantially increased due to temperature rise. When applying the current, Joule heating raises the temperature of the bimorph actuator. Due to the thermal expansion coefficient mismatch, the increased temperature then results in a difference in deformation between the Cr/Au layer and the underlying Si beam. In the experiment, the rotation angle Δθ of the mirror is defined as mentioned above shown in Figure 4. Figure 5a shows the optical microscope images taken at the applied currents of 0 and 0.04 A. Figures 5b shows the rotation angles of the mirrors *vs.* the applied current. Devices are measured at currents ranging from 0 A to 0.04 A. As expected, the device reaches the largest absolute value of the rotation angle at 0.04 A. With an applied current of 0.04 A, it is 1.69° and 1.35° for the low- and high-resistance devices, respectively. The maximum power consumptions occur at 0.04 A, and are 101 and 180 mW, respectively. Figure 5c shows the relations of the applied voltage (*i.e.*, voltage output from the power supply) *vs.* the applied current. The voltage increases with the applied current as expected. Figure 5d shows the total resistance *vs.* the applied current. The resistance increases with the applied current as mentioned earlier.

**Figure 6:**
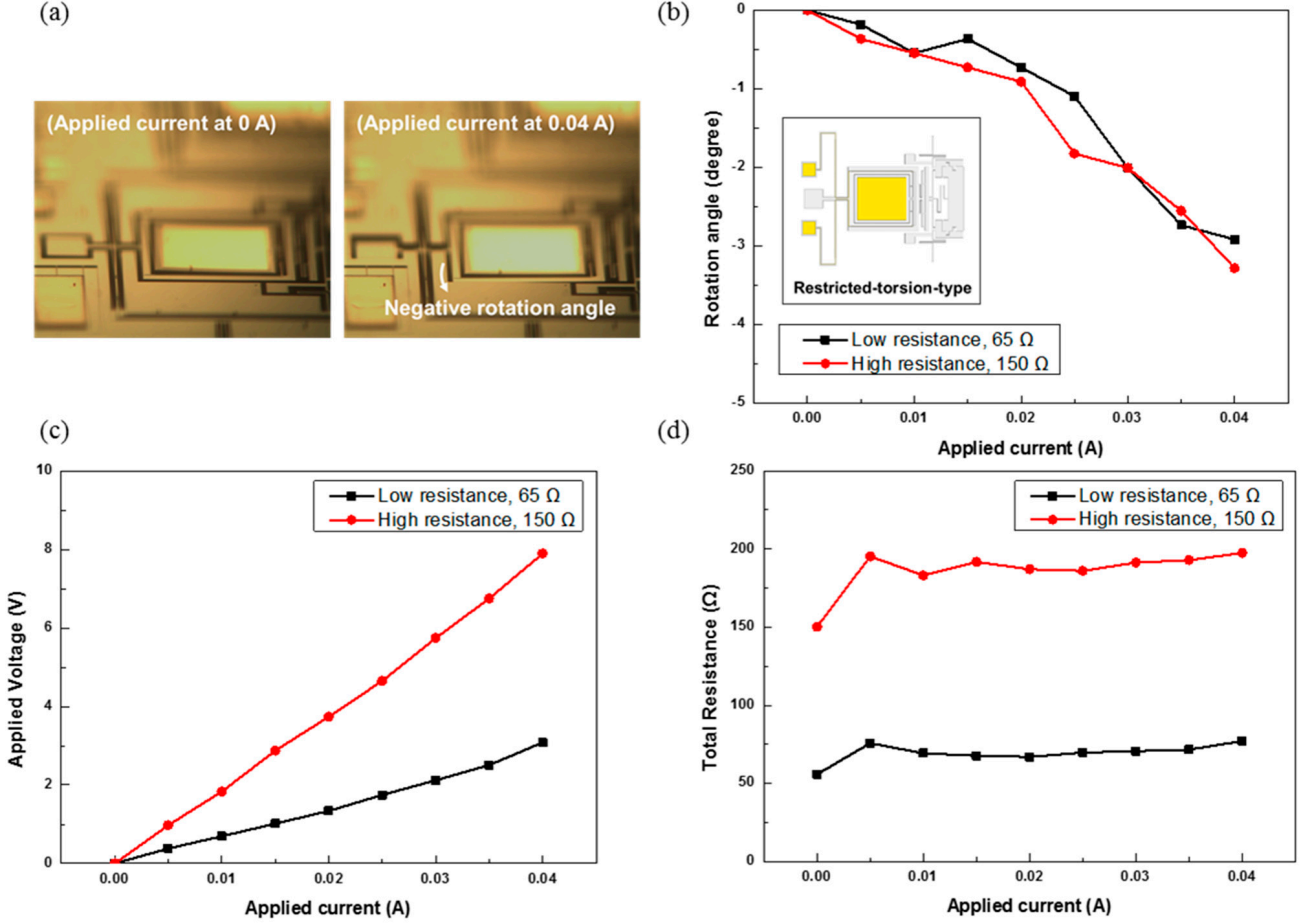
Experimental results for the restricted-torsion-type MEMS mirrors of different wire resistances: (**a**) optical microscope images; (**b**) rotation angles; (**c**) applied voltages; and (**d**) total resistances.

Figure 6 shows the experimental results for the restricted-torsion-type MEMS mirrors of different wire resistances. Figure 6a shows the optical microscope images at 0 and 0.04 A. Figure 6b shows the rotation angles of the mirrors *vs.* the applied current. At 0.04 A, the absolute value of the rotation angle is 2.91° and 3.28° for the low- and high-resistance devices, respectively. The power consumption at 0.04 A is 123 and 316 mW for the low- and high-resistance mirror, respectively. Figure 6c,d show the relations of the applied voltage and total resistance *vs.* the applied current. The applied voltage and total resistance both increase with the applied current.

**Figure 7:**
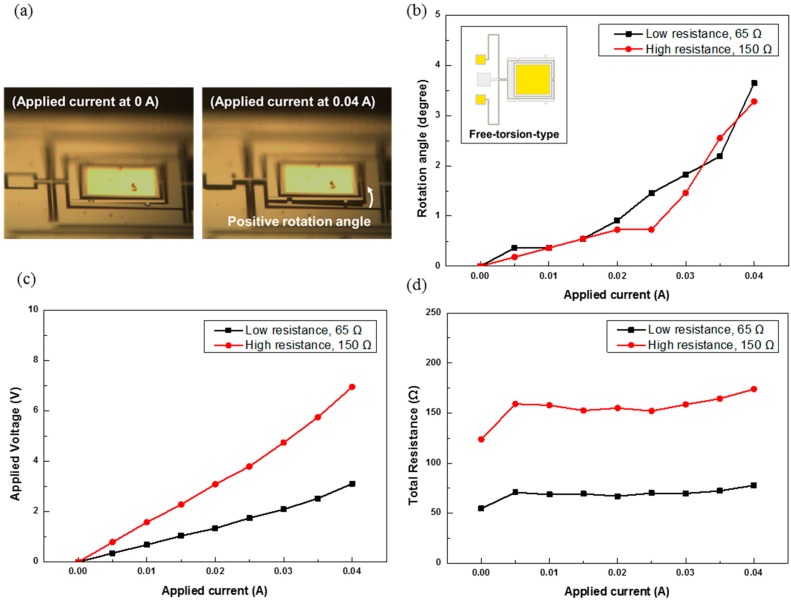
Experimental results for the free-torsion-type MEMS mirrors of different wire resistances: (**a**) optical microscope images; (**b**) rotation angles; (**c**) applied voltages; and (**d**) total resistances.

Figure 7 shows the experimental results for the free-torsion-type MEMS mirror of different wire resistances. Figure 7a shows the optical microscope image at 0 and 0.04 A. Figure 7b shows the rotation angles of the mirror *vs.* the applied current. The rotation angle at 0.04 A is 3.64° and 3.28° for the low- and high-resistance devices, respectively. The maximum power consumption occurs at 0.04 A; it is 124 and 278 mW for the low-and high-resistance mirrors, respectively. Figure 7c,d show the relations of the applied voltage and total resistance *vs.* the applied current. The total voltage and resistance both increase with the applied current.

**Figure 8:**
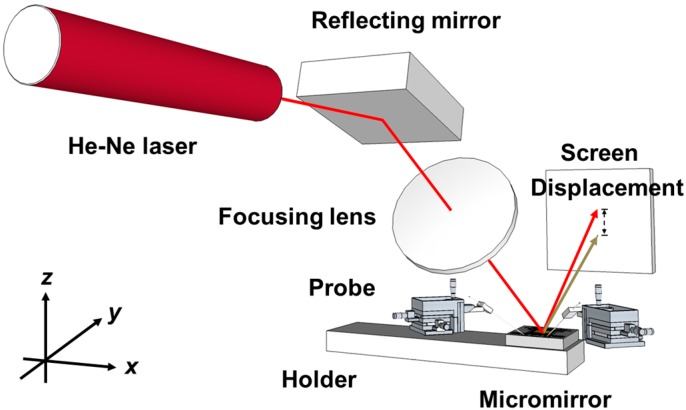
Schematic drawing of the experimental setup for tracing the light spot of the reflected laser beam.

**Figure 9:**
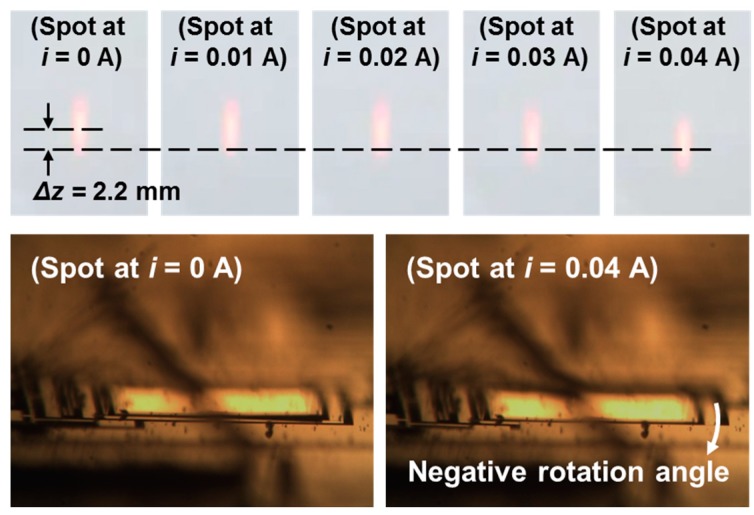
Trace of the light spot on a screen of the reflected laser beam from the restricted-torsion-type mirror. In addition, the mirror postures at *i* = 0 A and 0.04 A are shown.

Figure 8 shows a schematic diagram of the experimental setup for measuring laser beam displacement. The beam steering capability of the mirror was verified by shining a HeNe laser beam on the mirror and tracing the light spot of the reflected beam on a screen. The applied current is increased from 0 to 0.04 A with an increment of 0.01 A. Figure 9 shows the movement of the light spot and the mirror postures at 0 A and 0.04 A. The shift of the light spot is clearly observed.

## 4. Discussion and Conclusions

This study presents three different electrothermally actuated MEMS mirrors. A silicon-on-insulator MEMS process has been used for the fabrication of these micromirrors. In addition to a traditional bending structure, we also investigate the possibility of fabricating bimorph structures, which also serve as the torsion springs. At the maximum current of 0.04 A, the bending-type mirror has the lowest power consumption compared to the other two types of devices. The free-torsion-type mirror has the largest rotation range. The restricted-torsion-type mirror and free-torsion-type mirror have lower resonance frequencies than the bending-type mirror. 

These micromirrors, which are operated by electrothermal actuation, achieve decent angular movement. When an external electrical current of 0.04 A is applied, the bending-type, restricted-torsion-type, and free-torsion-type mirrors have rotation angles of 1.69°, 3.28°, and 3.64° (absolute values), respectively. It has been demonstrated that the devices can be fabricated with a simple fabrication process and work with a low driving voltage. They are potentially suitable for many optical applications.
